# Aggregation–Growth and Densification Behavior of Titanium Particles in Molten Mg-MgCl_2_ System

**DOI:** 10.3390/ma17122904

**Published:** 2024-06-13

**Authors:** Xin Yang, Kaihua Li, Jun Li, Zhuo Sheng, Ying Liu

**Affiliations:** 1 School of Materials Science and Engineering, Sichuan University, Chengdu 610065, China; yangxin15680824707@163.com (X.Y.);; 2 Key Laboratory of Vanadium and Titanium Resources Comprehensive Utilization, Panzhihua 617000, China; 3 Faculty of Metallurgical and Energy Engineering, Kunming University of Science and Technology, Kunming 650093, China; 4 Pangang Group Research Institute Co., Ltd., Panzhihua 617000, China

**Keywords:** titanium sponge, melting medium, aggregation and growth, pore structure, densification, kinetics

## Abstract

In this work, the preparation of titanium sponge by magnesium thermal method is regarded as the liquid-phase sintering process of titanium, and powder-metallurgy sintering technology is utilized to simulate the aggregation–growth and densification behavior of titanium particles in a high-temperature liquid medium (the molten Mg-MgCl_2_ system). It was found that compared with MgCl_2_, Mg has better high-temperature wettability and reduction effect, which promotes titanium particles to form a sponge titanium skeleton at lower temperature. The aggregation degree of titanium particles and the densification degree of a sponge titanium skeleton can be improved by increasing the temperature and the relative content of Mg in the melting medium. The kinetics study shows that with the increase in temperature, the porosity of the titanium particle aggregates and the sponge titanium skeleton decreases, and their density growth rate increases. With the extension of time, the aggregation degree of titanium particles and the densification degree of sponge titanium gradually increase. This work provides a theoretical reference for controlling the density of titanium sponge in industry.

## 1. Introduction

Titanium and titanium alloys have significant applications in aerospace, marine development, petrochemical industry, and clinical medical treatment. The efficient preparation of high-quality sponge titanium has become a concern [1]. In industrial production, the thermal reduction of titanium using Mg metal is an important method: under the protection of argon, titanium tetrachloride (TiCl_4_) is reduced by Mg metal to produce titanium sponge, and excess Mg and residual MgCl_2_ are then removed through vacuum distillation. The main reactions involved are as follows:

TiCl_4_(g) + 2Mg(l) = 2MgCl_2_(l) + Ti(s)

Akihiro Kishimoto and Tetsuya Uda [2] observed the in situ process from the addition of TiCl_4_ to the reaction between Mg and TiCl_4_, resulting in the formation of sponge titanium. They also studied the reaction mechanism of sponge titanium. Ch.R.V.S. Nagesh et al. [3] examined the stacking process of titanium particles in different stages of the magnesium thermal reduction reaction. Numerous reports have been published on the preparation of sponge titanium through the magnesium thermal reduction of TiCl_4_, and researchers have proposed various reaction mechanisms [4,5,6,7,8,9,10,11,12]. However, in general, the production process involves the following steps: the magnesium-based thermal reaction leading to the precipitation of fine titanium particles, gradual growth of these particles, aggregation and growth of titanium particles at high temperatures to form porous sponge titanium, and gradual densification of the porous sponge titanium in the molten Mg-MgCl_2_ system to form titanium sponge lumps [13,14,15,16,17,18]. However, the titanium sponge lumps produced using this method often exhibit uneven density distribution, particularly with a dense and hard core in the middle and lower parts. This results in a dense structure and coarse particles in titanium sponge, which can lead to composition segregation and structural defects in the preparation of titanium and titanium alloys. Consequently, controlling the particle size and pore structure of sponge titanium during production is a significant challenge in current research.

The process of producing sponge titanium through the magnesium thermal reduction reaction is highly complex, involving high temperatures, sealing, and kinetics imbalances [19,20]. Directly studying the laws governing the aggregation–growth and densification of titanium particles in industrial batch production is extremely difficult. In this work, we propose considering the process of titanium particle aggregation–growth to form sponge titanium as a liquid-phase sintering process of titanium particles in a molten Mg-MgCl_2_ system. Powder-metallurgy sintering technology is utilized to simulate the aggregation–growth and densification behavior of titanium particles in a high-temperature liquid medium. Therefore, this paper investigates the influence of medium composition on the aggregation–growth and densification behavior of titanium particles in the molten Mg-MgCl_2_ system. The findings provide a theoretical reference for controlling the particle size and pore structure of sponge titanium.

## 2. Materials and Methods

### 2.1. Materials

Pure Mg metal (purity ≥ 99.9%, provided by Qinghe Dingyuan Metal Products Co., Ltd., Xingtai, China), anhydrous MgCl_2_ (purity ≥ 99.9%, provided by Shanghai Aladdin Biochemical Technology Co., Ltd., Shanghai, China), and micron-sized titanium powder (purity ≥ 99.9%, D_50_ = 28.1 μm, provided by Zhejiang Yamei Nano Technology Co., Ltd., Jiashan, China) were used as raw materials.

### 2.2. Sample Preparation

Titanium powder, pure magnesium, and anhydrous magnesium chloride were added to a corundum crucible and thoroughly mixed before being placed into a vertical atmosphere furnace (SK-5-17Q, provided by FNS (Beijing) Electric Furnace Co., Ltd, Beijing, China) for sintering. In industrial production of titanium sponge, the reaction vessel experiences a temperature range from 750 °C to 1200 °C, with higher temperatures at the reaction center area [21,22]. As Mg undergoes thermal reduction, the molten Mg gradually reacts, while molten MgCl_2_ and sponge titanium are continuously generated. To simulate the aggregation–growth process of titanium particles, sintering temperatures were set at 800 °C, 900 °C, 1000 °C, 1100 °C, 1200 °C, and 1300 °C. The sintering media used were molten MgCl_2_ media and molten MgCl_2_:Mg (1:1) media. Before the sintering process, the equipment underwent three rounds of Ar gas purging. During the sintering process, Ar gas was continuously introduced at a flow rate of 1 L/min to prevent titanium particles from making contact with air and oxidizing at high temperatures. After sintering, the samples were subjected to vacuum distillation at 750 °C for 2 h to remove residual Mg and MgCl_2_ and obtain pure sponge titanium.

### 2.3. Characterization

Samples collected from different parts of the titanium sponge after sintering–distillation were analyzed. The high-temperature contact angles of the Mg-Ti and MgCl_2_-Ti systems were measured using a high-temperature contact angle measuring instrument (KRUSS DSAHT17C, Hamburg, Germany) to characterize the differences in wettability of different media on sponge titanium. The oxygen content of titanium particles and the composition of the oxide layer were characterized using a nitrogen–oxygen analyzer (ON-3000, Beijing, China) and X-ray photoelectron spectroscopy (XPS, K-Alpha, Waltham, MA, USA), respectively. The area percentage of titanium particles was measured using a metallographic microscope (Axio Observer A5m, Hanover, Germany) combined with Image-Pro Plus 6.0 software to assess the degree of aggregation of titanium particles. The average size change of titanium particle aggregates was measured using a laser particle size analyzer (Malvern Mastersizer 3000, Malvern, UK), and the growth behavior of aggregates in different media and at different temperatures was analyzed. The microstructure of the samples was observed using a field emission scanning electron microscope (ZEISS Sigma 500 FE-SEM, Oberkochen, Germany) to analyze the differences in the microstructure of titanium particles in different media. The total pore area, average pore size, and porosity of the samples were measured using a mercury intrusion meter (MicroActive AutoPore V 9600, Purchase, NY, USA) to analyze the changes in the pore structure of sponge titanium.

## 3. Results and Discussion

### 3.1. Influencing Factors in Liquid-Phase Sintering

**Figure 1:**
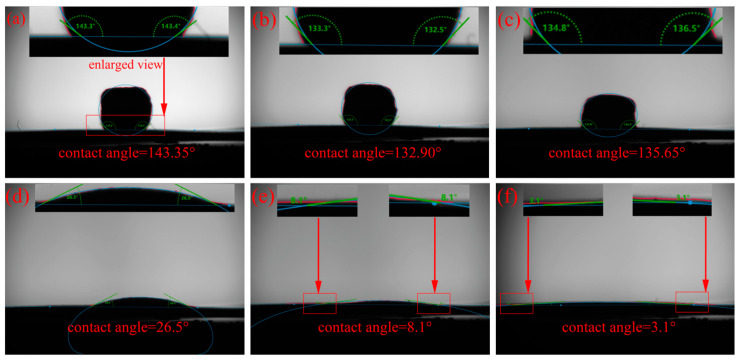
Photographs of the Mg-Ti contact angle test: (**a**) 25 °C, (**b**) 850 °C, (**c**) 900 °C, (**d**) holding the temperature at 900 °C for 1 min, (**e**) holding the temperature at 900 °C for 3 min, and (**f**) holding the temperature at 900 °C for 5 min.

**Figure 2:**
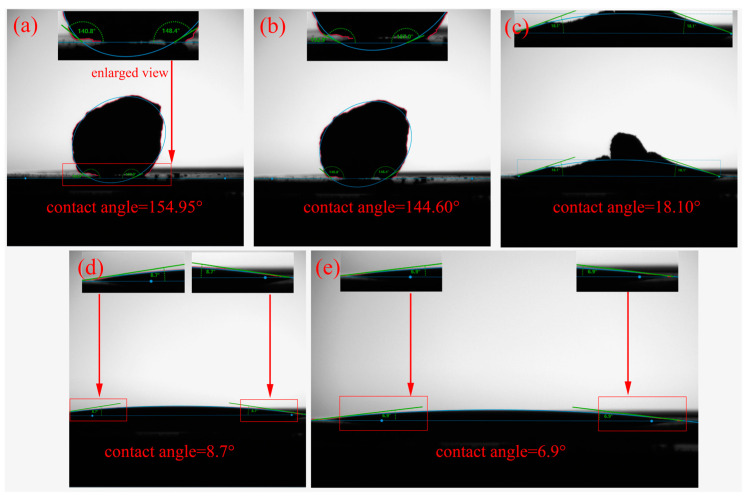
Photographs of the MgCl_2_-Ti contact angle test: (**a**) 25 °C, (**b**) 714 °C, (**c**) 900 °C, (**d**) holding the temperature at 900 °C for 1 min, and (**e**) holding the temperature at 900 °C for 5 min.

Figure 1 presents the results of the Mg-Ti wettability test. As the temperature rises from room temperature to 850 °C, the Mg remains solid and its shape remains unchanged. When the temperature reaches 900 °C, the Mg gradually melts. After holding at 900 °C for 1 min, the molten Mg starts to spread on the surface of the titanium substrate, resulting in a significant decrease in the contact angle. The contact angle of the molten Mg on the Ti sheet continues to decrease and eventually stabilizes at around 3.1° when the holding time is extended. Figure 2 shows the test results of the MgCl_2_-Ti wettability. As the temperature rises from room temperature to 714 °C, the shape of the MgCl_2_ remains unchanged as it has not yet melted. At 900 °C, the MgCl_2_ begins to melt, and its appearance undergoes a noticeable change. After holding at 900 °C for 1 min, the MgCl_2_ completely melts and spreads on the surface of the titanium substrate, leading to a significant decrease in the contact angle. When the holding time is extended to 5 min, the molten MgCl_2_ spreads completely on the titanium sheet, and the contact angle decreases to 6.9°. The contact angle of Mg-Ti is smaller than that of MgCl_2_-Ti, indicating that molten Mg has better wettability with Ti at high temperatures compared to molten MgCl_2_. This suggests that Ti atoms diffuse more easily in molten Mg [23,24]. On the other hand, better wettability of Mg/Ti means that molten Mg spreads more fully on the surface of titanium particles. The capillary force between adjacent titanium particles is greater, promoting the particles to come into contact with each other and form a sponge titanium framework, thereby accelerating the aggregation speed of titanium particles.

**Figure 3:**
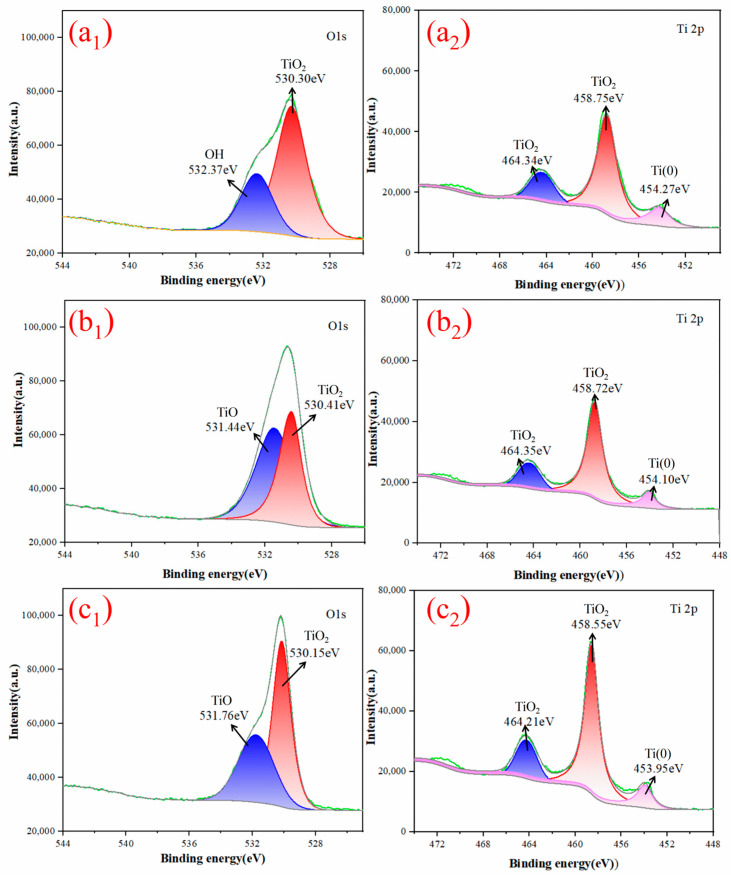
O1s XPS spectra of micron-sized titanium particles sintered at 1100 °C in (**a_1_**) a molten Mg media, (**b_1_**) a molten Mg:MgCl_2_ (1:1) media, and (**c_1_**) a molten MgCl_2_ media. Ti2p XPS spectra of micron-sized titanium particles sintered at 1100 °C in (**a_2_**) a molten Mg media, (**b_2_**) a molten Mg:MgCl_2_ (1:1) media, and (**c_2_**) a molten MgCl_2_ media.

Figure 3 displays the XPS spectra of O1s and Ti2p on the surface of titanium sponge sintered in different mediums. The O1s spectra exhibit two characteristic peaks, with binding energies of 531.0 eV and 531.3 eV, corresponding to the O element in TiO_2_ and TiO_2_, respectively. Additionally, the peak at 532.8 eV corresponds to the O element in bound water [25]. Thus, when titanium particles are sintered in a molten Mg medium, TiO_2_ is present on the surface of sponge titanium. After sintering the titanium particles in a molten Mg:MgCl_2_ (1:1) medium and a molten MgCl_2_ medium, the surface of the titanium particles contains TiO_2_ and TiO. The Ti2p spectrum can be fitted into three characteristic peaks, where the peaks at binding energies of 458.5 eV and 464.7 eV correspond to Ti^4+^ [26,27] in TiO_2_, and the peak at 453.86 eV corresponds to Ti (0) [28]. Following sintering in the three media, the characteristic peaks of TiO_2_ and elemental Ti (0) can be observed on the surface of titanium sponge, indicating that oxygen enters the titanium crystal lattice on the surface of the titanium particles and partially forms Ti-O bonds. In other words, there is an oxide layer on the surface of the titanium particles after liquid-phase sintering. This is because molten MgCl_2_ does not have a reducing effect, while titanium powder is highly reactive. During sintering, both the water introduced by MgCl_2_ and the brief contact of titanium powder with air will promote the oxidation of sponge titanium, forming TiO_2_ and TiO. Molten Mg can reduce the titanium oxides on the surface of sponge titanium, but due to the short reduction time, the reduction is not fully complete, leaving some residual TiO_2_.

**Figure 4:**
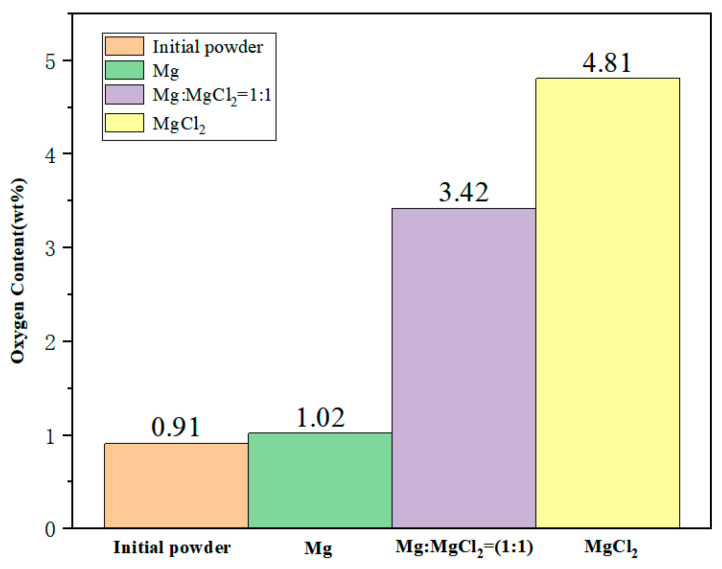
Variation in oxygen content of titanium after 2 h of sintering of titanium particles in different mediums at 1100 °C.

Figure 4 illustrates the changes in oxygen content of titanium particles after liquid-phase sintering in different mediums. In molten Mg, the oxygen content of titanium particles increases by only 0.11 wt% (growth rate: 12.1%). In the medium of molten Mg:MgCl_2_ = 1:1, the oxygen content increases by 2.51 wt% (growth rate: 275.8%). In molten MgCl_2_, the oxygen content increases significantly by 3.90 wt% (growth rate: 428.6%). The oxygen content in titanium particles increases with the increase in molten MgCl_2_ content. This is because a small amount of water-absorbing MgCl_2_, when melted, results in the adsorption of MgO on the surface of titanium particles. Furthermore, molten MgCl_2_ cannot effectively reduce the oxide layer on the surface of titanium particles. On the other hand, molten Mg can help reduce the increase in oxygen content on the surface of titanium particles during sintering. However, MgO, a by-product of the reduction process, is difficult to eliminate from the surface of titanium particles and remains within the particles after distillation. As a result, compared to other mediums, the titanium particles sintered in molten Mg show the smallest increase in oxygen content.

### 3.2. Aggregation Behavior of Titanium Particles

**Figure 5:**
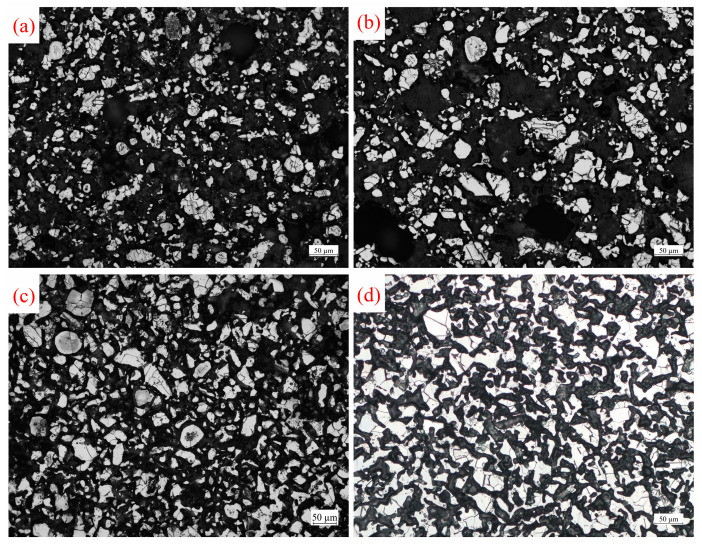
Metallographic photographs of titanium particles after 2 h of aggregating in molten MgCl_2_ at different temperatures: (**a**) 800 °C, (**b**) 900 °C, (**c**) 1000 °C, and (**d**) 1100 °C.

Figure 5 depicts a metallographic photograph of titanium particles aggregated in a molten MgCl_2_ medium at different temperatures for 2 h. In the metallographic diagrams, white area represents titanium particles or aggregates, while black area represents pores. As the temperature increases, the wettability between the liquid phase and the solid phase improves [29,30]. The molten medium can spread more thoroughly over the surface of the titanium particles, enhancing the capillary forces between them and accelerating their aggregation. At 800 °C (Figure 5a), the number of titanium particles in the medium per unit area is small and sparsely distributed, accounting for 17.2% of the area. As the temperature increases to 900 °C and 1000 °C (Figure 5b,c), the number of titanium particles in the medium per unit area begins to increase, and the degree of aggregation gradually intensifies. The area proportion occupied by titanium particles reaches 20.0% and 31.4%, respectively. There are still gaps between adjacent titanium particles, indicating that they have not yet fully connected with each other, and the titanium particles remain in the aggregation stage. At 1100 °C (Figure 5d), the diffusion of titanium atoms becomes more pronounced. The area proportion of titanium particles further increases to 35.4%, and adjacent titanium particles become connected, resulting in the aggregation of titanium particles and the formation of a sponge titanium skeleton.

**Figure 6:**
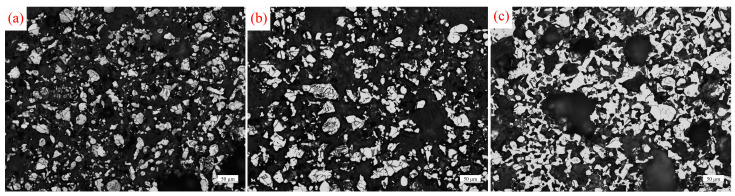
Metallographic photographs of titanium particles after 2 h of aggregating in molten Mg:MgCl_2_ (1:1) at different temperatures: (**a**) 800 °C, (**b**) 900 °C, and (**c**) 1000 °C.

Figure 6 presents a metallographic photograph of titanium particles aggregated in a molten Mg:MgCl_2_ (1:1) medium at different temperatures for 2 h. The wettability between Mg and Ti is better than that between MgCl_2_ and Ti. In molten Mg, the capillary force between titanium particles is stronger, accelerating the aggregation speed of titanium particles. Additionally, molten Mg has a reducing effect, which reduces the oxide layer on the surface of titanium particles and weakens the obstacle to the diffusion of titanium atoms. This further accelerates the aggregation of titanium particles, allowing the formation of a sponge titanium skeleton at a lower temperature. Compared to the molten MgCl_2_ medium, the increased content of molten Mg in the mixed medium accelerates the aggregation rate of titanium particles. At 800 °C (Figure 6a), the proportion of titanium particles is 19.7% (an increase of 2.5% compared to Figure 5a), and their distribution remains relatively dispersed. At 900 °C (Figure 6b), titanium particles continue to gather, and their quantity increases, resulting in an area proportion of titanium particles of 21.4%. Upon reaching 1000 °C (Figure 6c), the area ratio of titanium particles reaches 32.2%. Adjacent titanium particles are connected to each other, leading to the aggregation of titanium particles and the formation of a sponge titanium skeleton.

**Figure 7:**
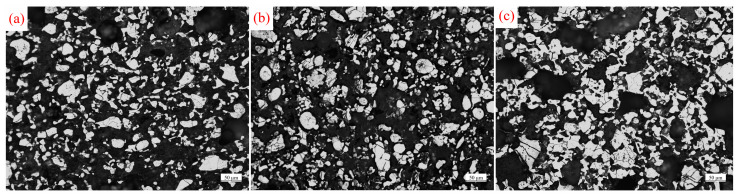
Metallographic photographs of titanium particles after 2 h of aggregating in molten Mg at different temperatures: (**a**) 800 °C, (**b**) 900 °C, and (**c**) 1000 °C.

Figure 7 displays metallographic photographs of titanium particles after liquid-phase aggregating in molten Mg at different temperatures for 2 h. The content of Mg in the molten medium continues to increase, and the wetting and reducing effects of the molten medium are strengthened, which accelerates the aggregation speed of titanium particles. At 800 °C and 900 °C (Figure 7a,b), titanium particles continue to aggregate, accounting for 23.2% and 26.8%, respectively, which is significantly higher compared to the other two media. At 1000 °C (Figure 7c), the aggregation degree of titanium particles increases significantly, resulting in an area proportion of 34.3%. The titanium particles are interconnected, forming an aggregated structure resembling a sponge titanium skeleton. As the content of molten Mg in the medium increases, the aggregation speed of titanium particles also increases, leading to a higher area proportion at the same temperature.

**Figure 8:**
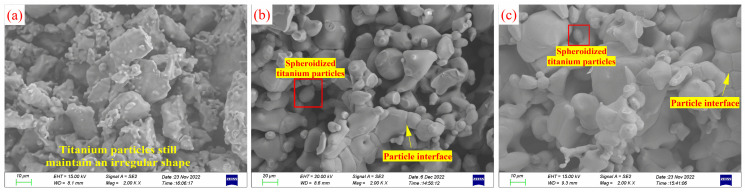
SEM images of titanium particles aggregated in different mediums for 2 h at 900 °C: (**a**) molten MgCl_2_ medium, (**b**) molten MgCl_2_:Mg (1:1) medium, and (**c**) molten Mg medium.

Figure 8 depicts the micrograph of titanium particles aggregated in different media at 900 °C for 2 h. In the case of aggregating in a molten MgCl_2_ medium, titanium exhibits no solubility, resulting in the preservation of the original irregular morphology of titanium particles. Fine titanium particles and sharp edges of titanium particles cannot be dissolved, leading to their accumulation on the surface of larger particles (Figure 8a). According to the Ti-Mg binary phase diagram [31,32], titanium possesses certain solubility in molten Mg, which increases gradually with temperature. During aggregating, particles with high surface energy and sharp edges tend to dissolve first [33]. They subsequently nucleate and precipitate on the surface of larger particles or defective areas, causing the disappearance of smaller particles and the growth and spheroidization of larger particles. This process, known as Oswald ripening, can be observed in Figure 8a,b (highlighted by the red boxes). Consequently, the fine particles dissolve and vanish, while the sharp edges of larger particles continuously dissolve and spheroidize. The Oswald ripening phenomenon of titanium particles in molten Mg accelerates the diffusion of titanium atoms through the continuous dissolution and reprecipitation of titanium. This enhances solid-phase mass transfer, accelerating the aggregation of titanium particles. The aggregation of titanium particles leads to the formation of more particle interfaces, as shown in Figure 8c. The microstructure of titanium particles after aggregation is similar to that of mesoporous hematite/alumina nanocomposites [34] and iron oxide nanochains coated with silica [35], as reported by Marin Tadic. The particles contact and adhere to each other to form a ‘spongelike’ structure, with numerous pores between the particles.

**Figure 9:**
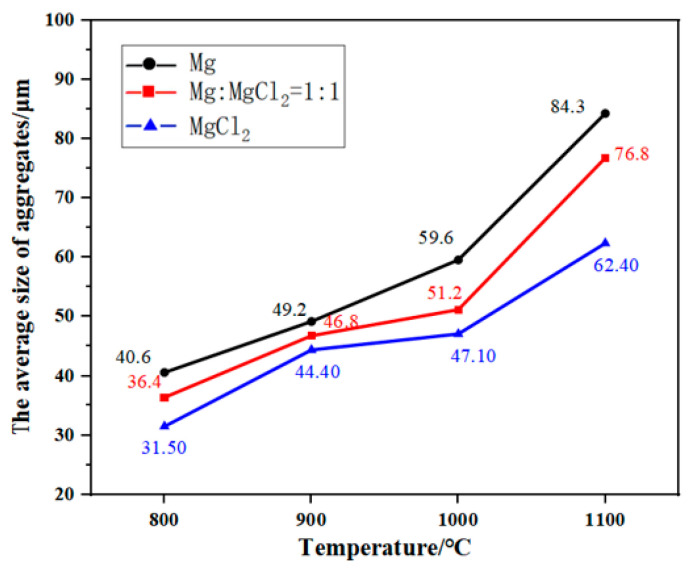
Variation in particle size with temperature after aggregating of titanium particles in different mediums for 2 h.

Figure 9 illustrates the changes in the average size of titanium particle aggregates with temperature after aggregating in various media for 2 h. The average size of titanium particle aggregates increases with temperature in all three mediums. In the case of aggregating in molten MgCl_2_, small particles accumulate on the surface of larger particles. As the temperature rises, the number and size of aggregates formed by small particles increase. In the medium containing molten Mg, titanium particles undergo continuous growth through Oswald ripening. Simultaneously, the reduction of the oxide layer on the particle surfaces by molten Mg accelerates mass transfer between titanium particles, facilitating faster particle aggregation and the formation of interfaces. With increasing temperature, the coarsening rate of particles intensifies, resulting in a greater number of particles in contact with each other and larger aggregate sizes.

The average size of aggregates is largest in molten Mg and smallest in molten MgCl_2_. This is attributed to the formation of a liquid-phase film that covers the surface of titanium particles during aggregating. Capillary forces drive the liquid phase to fill the gaps between particles, promoting particle aggregation, increasing the contact area between particles, enhancing mass transfer, and facilitating easier diffusion of atoms between adjacent titanium particles through the liquid-phase channels. Compared to molten MgCl_2_, molten Mg exhibits better wetting properties toward titanium and stronger capillary action [36], which promotes particle aggregation and leads to larger aggregate sizes. Additionally, molten Mg reduces the oxide layer on the surface of titanium particles, accelerating mass transfer between particles and promoting particle aggregation. On the other hand, molten MgCl_2_ cannot reduce the oxide film on the surface of titanium particles, thereby inhibiting titanium atom diffusion and impeding particle aggregation. Therefore, a higher content of molten Mg in the medium facilitates greater particle aggregation and faster growth rates of aggregates.

The process of titanium particle aggregation involves the precipitation of dissolved titanium atoms from molten Mg, the nucleation of titanium atomic clusters to fill the pores, and the nucleation and growth process after particle contact, thereby reducing the porosity of titanium particle aggregates. The nucleation and growth rates of titanium particles during the aggregation process are uniform and occur at a certain rate, and this process, controlled by nucleation and growth dynamics, can be effectively explained by the Johnson–Mehl–Avrami (JMA) model [37,38,39]. Using the JMA model, the aggregation process of titanium particles at different temperatures and times can be studied to explain the kinetics of the aggregation process. Its expression formula is as follows:

ξ = 1 − exp (−kt^n^) (1)

where “ξ” presents the percentage of phase proportion, which can be expressed by the relative density of aggregates of titanium particles; “k” presents a kinetics constant dependent on temperature (T); “t” presents time; and “n” presents Avrami index, which reflects kinetics of the aggregation process of titanium particles. The evolution of Equation (1) yields Equation (2), where “1 − ξ” can be expressed as the porosity of aggregates of particles.

ln(1/(1 − ξ)) = lnk + nlnt(2)

**Figure 10:**
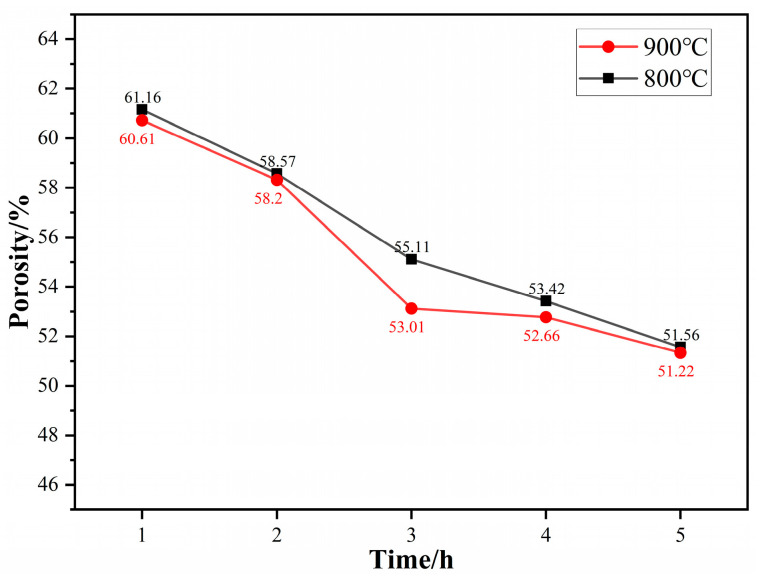
Porosity of titanium particles after aggregation at different temperatures and different times in MgCl_2_:Mg (1:1).

Figure 10 illustrates the porosity of titanium particles after aggregation at different temperatures and different times in MgCl_2_:Mg (1:1). At room temperature, the bulk density of raw titanium powder is 1.26 g/cm^3^, and the calculated porosity is 72.0%. After aggregating in MgCl_2_:Mg (1:1) at 800 °C for 1 h, the porosity of titanium aggregates rapidly decreases to 61.16%. At the same aggregation time, with the increase in temperature, the wettability, reduction, and dissolution–precipitation effect of molten Mg are enhanced, and the diffusion of Ti atoms is also strengthened, which promotes the aggregation process of the titanium particles. After the titanium particles are aggregated at 900 °C for 1 h, the porosity of the particles decreases to 60.61%. The porosity of the aggregates decreases with the increase in temperature and time.

**Figure 11:**
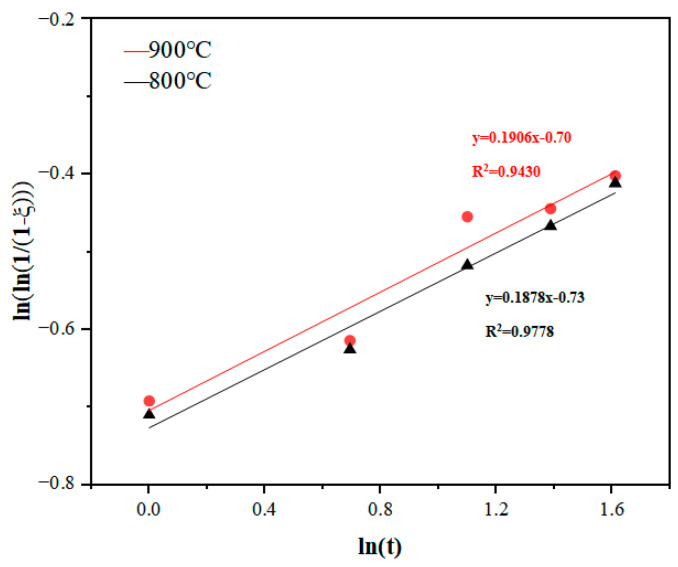
Fitting curves of the relationship between ln(1/(1 − ξ)) and lnt at different temperatures.

Figure 11 illustrates the fitting curves of the relationship between ln(1/(1 − ξ)) and lnt at different temperatures, which are plotted according to the data in Figure 10. As can be seen from Figure 11, the relationship between ln(1/(1 − ξ)) and lnt basically presents a linear relationship at different temperatures. According to Equation (2), the Avrami index “n” and the kinetics constant “k” can be calculated by the slope and intercept of the fitted lines in the figure, respectively, and their values are shown in Table 1.

**Figure 12:**
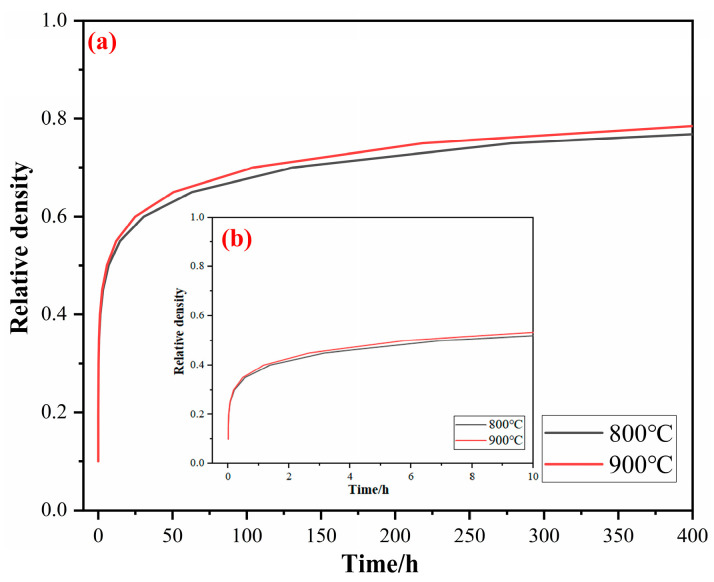
Kinetics curves of titanium particles aggregation at different temperatures in MgCl_2_:Mg (1:1): (**a**) in the range of 0~400 h and (**b**) partially enlarged detail in the range of 0~10 h.

According to the kinetics equation, the corresponding kinetics curve is shown in Figure 12. At room temperature, the bulk density of raw titanium powder is 1.26 g/cm^3^, and its relative density is 28.0%. In this paper, it is considered that the initial relative density in the kinetics curve is 28.0% (i.e., ξ = 0.28). Figure 12a shows that at the same time, the higher the temperature, the higher the density of the titanium particle aggregates and the lower the porosity. This is because as the temperature increases, the diffusion of titanium atoms speeds up. Simultaneously, the wettability, reducibility, and solubility of molten Mg improve, promoting the diffusion of titanium atoms, accelerating particle aggregation, and reducing aggregate porosity. However, when the temperature is low, the activation energy for atomic diffusion is insufficient, and the density of the aggregates remains close to 80% even after 400 h of aggregation. Figure 12b indicates that the porosity of the aggregates increases rapidly from 0 to 3 h and then increases slowly with prolonged time. This is because during the initial 0 to 3 h, molten Mg fully dissolves the titanium in the concave–convex or angular parts of the surface, reaching dynamic equilibrium, while also essentially completing the reduction of the oxide layer on the titanium particle surfaces. Over time, the dissolution–precipitation effect significantly weakens the filling of pores, thereby slowing down the aggregation rate of titanium particles. From the kinetics curves, it can be observed that during the aggregation of titanium particles, increasing the temperature from 800 °C to 900 °C results in only a slight increase in the relative density of the aggregates at the same time. However, with prolonged time, the relative density increases significantly. This indicates that time has a more significant effect on the relative density and porosity of titanium sponge during the aggregation process.

### 3.3. Densification Behavior of Sponge Titanium

**Figure 13:**
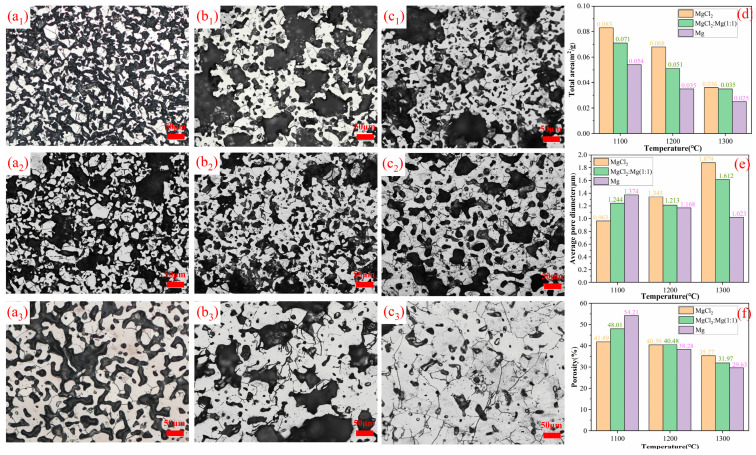
Densification behavior of sponge titanium in three different mediums and temperature: (**a_1_**–**a_3_**) represent the metallographic diagrams of sponge titanium densified in molten MgCl_2_ at 1100 °C, 1200 °C, and 1300 °C, respectively; (**b_1_**–**b_3_**) represent the metallographic diagrams of sponge titanium in MgCl_2_:Mg (1:1) densified at 1100 °C, 1200 °C, and 1300 °C, respectively; (**c_1_**–**c_3_**) represent the metallographic diagrams of sponge titanium densified in molten Mg at 1100 °C, 1200 °C, and 1300 °C, respectively; the length of the scale in the photomicrograph represents 50 μm; (**d**–**f**) represent the variations in total pore area, average pore size, and porosity of the titanium sponge samples.

Figure 13 illustrates the densification behavior of titanium sponge in three different media. In the metallographic diagrams, white area represents the titanium sponge skeleton, while black area represents the pores. At 1100 °C, the wetting, dissolution, and reduction caused by molten Mg accelerate the densification of titanium sponge. As a result, the area ratio of the titanium sponge skeleton is the smallest in molten MgCl_2_ and the largest in molten Mg, measuring 36.2% (in molten MgCl_2_, Figure 13(a_1_)) and 38.6% (in MgCl_2_:Mg (1:1), Figure 13(b_1_)), respectively. At 1200 °C, compared to the values at 1100 °C, the area proportion of the titanium sponge skeleton increases to 38.9% (in molten MgCl_2_, Figure 13(a_2_)), 47.5% (in MgCl_2_:Mg (1:1), Figure 13(b_2_)), and 52.5% (in molten Mg, Figure 13(c_2_)), respectively. At 1300 °C, the area proportion of the titanium sponge skeleton further increases to 52.3% (in molten MgCl_2_, Figure 13(a_3_)), 60.0% (in MgCl_2_:Mg (1:1), Figure 13(b_3_)), and 78.7% (in molten Mg, Figure 13(c_3_)).

The wettability and reducibility of molten Mg promote the diffusion of titanium atoms. Simultaneously, titanium dissolves in molten Mg at high temperatures. The precipitation of titanium clusters at the pores reduces the surface energy of the system due to the high surface energy at the pores. This promotes the nucleation and precipitation of titanium atoms at the pores, allowing for local filling of macropores and rapid reduction in the pore area. In molten MgCl_2_, titanium cannot fill the pores through dissolution–precipitation, hindering pore shrinkage. Consequently, the total pore area of the titanium sponge is larger than that in the molten Mg medium (Figure 13d). At 1100 °C, the dissolution of titanium particles in molten Mg causes their shape to become spheroidized. This change promotes alterations in pore shape and increases the pore size between the skeleton structures, resulting in the largest average pore diameter of the titanium sponge. As the temperature rises, the solubility of titanium in molten Mg increases, leading to stronger precipitation and filling of titanium atoms and a gradual decrease in the average pore size of the titanium sponge. In molten MgCl_2_, the fine pores in the titanium sponge gradually shrink and disappear, while molten MgCl_2_ tends to remain in the large pores of the titanium sponge, hindering shrinkage and causing a continuous increase in the average pore size of the titanium sponge (Figure 13e). At 1100 °C, the spheroidization of titanium particles in molten Mg alters the pore shape between the skeleton structures and weakens the mechanical locking between particles, resulting in a high porosity of the titanium sponge. In molten MgCl_2_, the titanium sponge continuously densifies through atomic diffusion, leading to a continuous reduction in porosity. As the temperature rises, the diffusion of titanium atoms becomes the dominant factor. Compared to molten MgCl_2_, the titanium sponge exhibits higher density in molten Mg, with the lowest porosity, while the highest porosity is observed in MgCl_2_.

**Figure 14:**
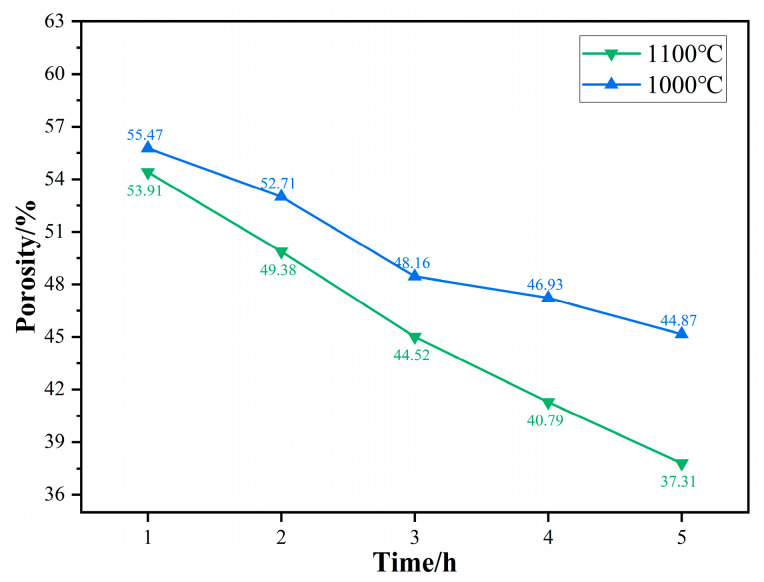
Porosity of titanium sponge after densification at different temperatures and different times in MgCl_2_:Mg (1:1).

Figure 14 illustrates the porosity variation of titanium sponge in MgCl_2_:Mg (1:1) after densification at different temperatures and at different times. With the extension of time, the mass transfer between solid titanium is more sufficient, and the number of titanium clusters precipitated in molten Mg increases, enhancing the filling effect on the pores in the skeleton and continuously reducing the porosity of the sponge titanium. The porosity of titanium sponge decreased from 55.47% to 44.87% at 1000 °C and from 53.91% to 37.31% at 1100 °C. For the same time period, the porosity of titanium sponge at 1100 °C is lower than that at 1000 °C. This is because, at higher temperatures, the diffusion of titanium atoms and grain growth occur more rapidly, promoting the densification of the titanium sponge. Additionally, the strengthened dissolution–precipitation effect of molten Mg on titanium increases the filling effect on the pores, leading to a reduction in the porosity of the titanium sponge skeleton.

**Figure 15:**
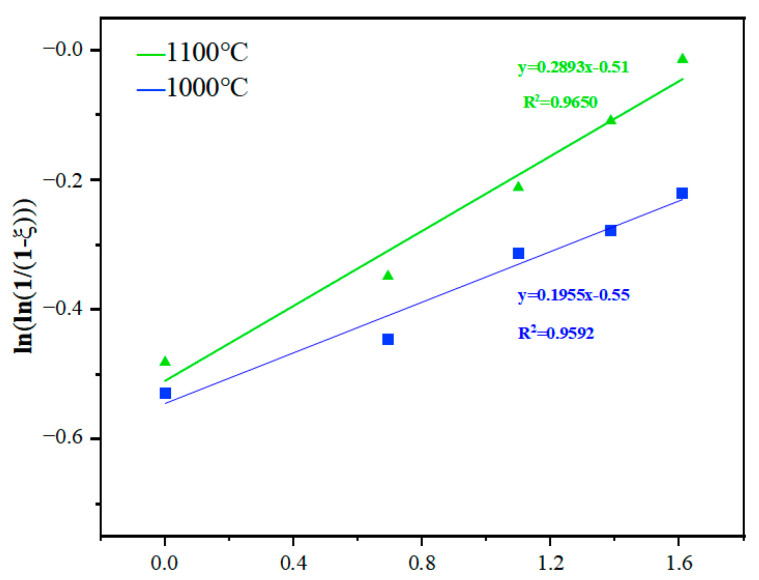
Fitting curves of the relationship between ln(1/(1 − ξ)) and lnt at different temperatures.

The Avrami formula is also used to study the kinetics of sponge titanium skeleton densification. The “ξ” in Equation (1) can be used to represent the relative density of the titanium sponge skeleton. The curves for ln(1/(1 − ξ)) and lnt are fitted by using the same method as in Section 3.2 and are shown in Figure 15. As can be seen from Figure 15, the relationship between ln(1/(1 − ξ)) and lnt basically presents a linear relationship at different temperatures. According to Equation (2), the Avrami index “n” and the kinetics constant “k” are calculated and listed in Table 2.

**Figure 16:**
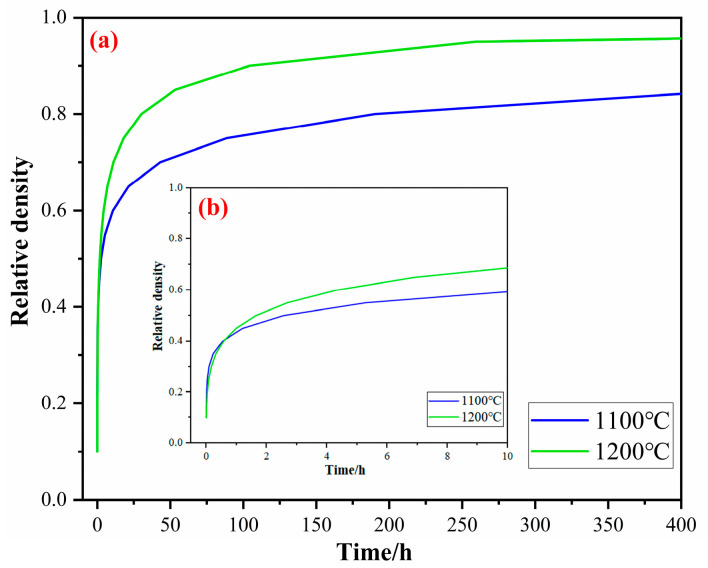
Kinetics curve of the titanium sponge skeleton densification at different temperatures in MgCl_2_:Mg (1:1): (**a**) in the range of 0~400 h and (**b**) partially enlarged detail in the range of 0~10 h.

Figure 16 shows the kinetics curve of the titanium sponge skeleton at different temperatures in MgCl_2_:Mg (1:1). The kinetics curve exhibits a rapid rise from 0 h to 3 h, and then shows a slow increase from 3 h to 80 h and continues to extend the time. The kinetics curve essentially remains stable, indicating that the densification process has reached its limit. In the early stage of densification, there are a lot of pores in the titanium sponge. Through the diffusion of titanium atoms and the filling of the precipitated titanium atoms, the pores in the titanium sponge shrink and close rapidly, leading to a rapid decrease in porosity. In the latter stage of densification, as the number of pores decreases, it becomes difficult for the remaining pores to continue shrinking without external force, resulting in a slower decrease in porosity. With the increase in temperature, the diffusion of titanium atoms is accelerated, and the solid-phase mass transfer rate of Ti is increased, resulting in the increase in the limit value of the kinetics curve, indicating that the maximum degree of densification is increased.

From the kinetics curves of the densification process, it is evident that the trend of relative density increase in the sponge titanium is similar over time. But at 1200 °C, the sponge titanium exhibits a significantly higher relative density. This shows that during the densification stage of the sponge titanium, temperature is the main factor affecting its relative density and porosity.

## 4. Conclusions

This paper investigates the effects of the composition of a high-temperature liquid medium (the molten Mg-MgCl_2_ system) on the aggregation–growth and densification behavior of titanium particles, coming to the following conclusions:

1. Compared to molten MgCl_2_, molten Mg demonstrates superior reduction, dissolution, and wetting effects, which promote the diffusion of titanium atoms, facilitate the aggregation of titanium particles, and accelerate the formation of the titanium sponge skeleton. In the molten MgCl_2_ medium, titanium particles aggregate and grow at 1100 °C, resulting in the formation of the titanium sponge skeleton. In the molten Mg medium and MgCl_2_:Mg (1:1) medium, titanium particles aggregate and grow at 1000 °C, leading to the formation of the titanium sponge skeleton. With the increase in temperature, the wettability, reducibility, and solubility of the molten medium are improved, promoting the diffusion of titanium atoms, strengthening the capillary forces between titanium particles, and accelerating the aggregation of titanium particles.
2. The Oswald ripening mechanism promotes the coarsening of titanium particles, and the reduction in molten Mg accelerates the aggregation of particles, causing the aggregates to grow rapidly in molten Mg. When aggregated in molten MgCl_2_, the aggregate size increases through the gathering of small particles on the surface of large particles, resulting in a slower growth rate.
3. With an increase in the relative content of molten Mg in the medium, the diffusion of titanium atoms is promoted, and the solid titanium’s mass transfer is accelerated, which benefits the densification of the titanium sponge. At different temperatures, the area ratio of the titanium sponge skeleton is highest in molten Mg, while the total pore area, average pore size, and porosity are lower compared to the other media.
4. Kinetics studies show that the porosity of titanium particle aggregates and the porosity of the titanium sponge skeleton decrease with increasing temperature at the same time, and the limiting value of the relative density increases. The aggregation rate of titanium particles and the densification rate of titanium sponge were higher at 0~3 h. From 3 h to 80 h, the aggregation rate of titanium particles and the densification rate of titanium sponge gradually decreased and continued to extend the time. The relative density basically remained unchanged, and the density of aggregates and titanium sponge reached the limit.

In summary, increasing the Mg content in the melting medium and raising the temperature will accelerate the aggregation–growth and densification of titanium particles, resulting in low-quality sponge titanium with low porosity and high compactness. Therefore, in industrial production, the aggregation–growth rate and densification degree of titanium particles can be delayed by controlling the temperature (enhancing heat dissipation, etc.) and adjusting the composition of the melting medium (reducing the emission of MgCl_2_, etc.) to obtain high-quality titanium sponge with a loose structure.

## Figures and Tables

**Table 1:** The kinetics equation of the aggregation of titanium particles at different temperatures.

Temperature	Kinetics Formulas	1 − ξ				
		1 h	2 h	3 h	4 h	5 h
800 °C	ξ = 1 − exp(−0.4819t^0.1878^)	0.6116	0.5857	0.551	0.5342	0.5156
900 °C	ξ = 1 − exp(−0.4966t^0.1906^)	0.6061	0.5820	0.5301	0.5266	0.5122

**Table 2:** Kinetics formulas at different temperatures.

Temperature	Kinetics Formulas	1 − ξ				
		1 h	2 h	3 h	4 h	5 h
1000 °C	ξ = 1 − exp(−0.5769t^0.1955^)	0.5547	0.5271	0.4816	0.4693	0.4487
1100 °C	ξ = 1 − exp(−0.6005t^0.2893^)	0.5391	0.4938	0.4452	0.4079	0.3731

## Data Availability

The original contributions presented in the study are included in the article, further inquiries can be directed to the corresponding author.
